# Oncolytic Virotherapy of Canine and Feline Cancer 

**DOI:** 10.3390/v6052122

**Published:** 2014-05-16

**Authors:** Ivaylo Gentschev, Sandeep S. Patil, Ivan Petrov, Joseph Cappello, Marion Adelfinger, Aladar A. Szalay

**Affiliations:** 1 Department of Biochemistry, University of Wuerzburg, Wuerzburg D-97074, Germany; E-Mails: sandeep.patil@stud-mail.uni-wuerzburg.de (S.S.P.); ivan.petrov@uni-wuerzburg.de (I.P.); marion.adelfinger@biozentrum.uni-wuerzburg.de (M.A.); 2 Genelux Corporation, San Diego Science Center, San Diego, CA 92109, USA; E-Mail: Joseph.Cappello@genelux.com; 3 Department of Radiation Medicine and Applied Sciences, Rebecca and John Moores Comprehensive Cancer Center, University of California, San Diego, La Jolla, CA 92093, USA

**Keywords:** cancer, canine and feline cancer therapy, oncolytic virus, oncolysis

## Abstract

Cancer is the leading cause of disease-related death in companion animals such as dogs and cats. Despite recent progress in the diagnosis and treatment of advanced canine and feline cancer, overall patient treatment outcome has not been substantially improved. Virotherapy using oncolytic viruses is one promising new strategy for cancer therapy. Oncolytic viruses (OVs) preferentially infect and lyse cancer cells, without causing excessive damage to surrounding healthy tissue, and initiate tumor-specific immunity. The current review describes the use of different oncolytic viruses for cancer therapy and their application to canine and feline cancer.

## 1. Introduction

Cancer is among the top fatal diseases in domestic and feral dogs and cats [1,2,3,4,5]. Incidence of canine or feline cancer ranges from 1% to 2% and cancer currently accounts for about half of the deaths of domestic animals older than 10 years [1,6,7]. The most common forms of cancer in dogs and cats are skin, lymphoma, mammary, bone, connective tissue, and oral cancers [1,7]. The traditional and established methods for pet cancer treatment include surgery, radiation therapy, chemotherapy, hyperthermia and photodynamic therapy. However, the available treatment options for pet patients with advanced-stage disease are limited and the prognosis for such patients is very poor. Therefore, developing novel therapies, which may also work synergistically in combination with the conventional treatment options, is crucial. 

One promising new therapeutic approach is oncolytic virotherapy. Oncolytic viruses (OVs) exhibit selective viral replication in tumors and metastases resulting in the killing of cancer cells and the initiation of tumor-specific immunity [8,9]. Several OVs including adenovirus, herpes simplex virus, vaccinia virus, Seneca Valley virus and reovirus are currently in human clinical trials, the most advanced of which is in Phase III [10,11]. In addition, in China, the oncolytic adenovirus H101 has been approved for combination therapy of human cancer patients [12]. 

In contrast to the progress of human oncolytic virotherapy, there are very few clinical trials using OVs for canine or feline cancer patients [13,14]. Since many forms of canine or feline neoplasms resemble their human counterparts in histological appearance, tumor genetics, biologic behavior, pathologic expression, recognized risk factors and response to therapy [15,16,17,18], it is reasonable to expect that the human clinical protocols will transfer directly to the treatment of pet cancer patients. There are strong similarities between naturally occurring human and canine cancers including colorectal carcinoma, fibrosarcoma, osteosarcoma, soft tissue sarcoma, Non-Hodgkin and Burkitt lymphomas and small lymphocytic lymphoma [19]. Several types of feline neoplasms such as squamous cell carcinoma and mammary carcinoma also show similarities in tumor biology, expression patterns and prognosis with human head and neck squamous cell carcinoma and a wide subset of human breast cancers [20,21]. As OVs show continuous promise in clinical application for treatment of many cancer types in human patients and considering the high similarity and increasing frequency of these cancers in pets, we believe that oncolytic virotherapy could soon be a reality also in veterinary medicine. Moreover the data from canine studies are more reliable and may be helpful in designing human clinical trials. The translation of oncolytic virotherapy from dogs to humans and the reverse could be a two-way street for development of drugs.

This review describes the most common classes of OVs and their progress in preclinical studies with canine and feline cancers. Presented are also the challenges and the major obstacles to the optimal practice of oncolytic virotherapy in pet cancer patients. 

## 2. Oncolytic Virotherapy for Canine and Feline Cancers

Many wild type or recombinant viruses have been tested as oncolytic agents for treatment of canine or feline cancer. They include human and canine adenoviruses, canine distemper virus (CDV), reovirus and members of the poxvirus family such as vaccinia virus, recombinant canary poxvirus (ALVAC), NYVAC (derived from the Copenhagen vaccinia strain) and myxoma virus. 

### 2.1. Adenoviruses

Adenoviruses have been widely characterized for oncolytic therapy in humans, but beyond this they have the ability to infect a broad range of cell types across many species [22,23]. For this reason, human and canine adenoviruses have been used as therapeutic agents for canine and feline cancers (Table 1). For example, human adenovirus 5 (Ad5) productively replicates in canine osteosarcoma, canine melanoma and canine mammary carcinoma cells [24,25]. Several Ad5-based adenoviral vectors expressing different genetic or molecular factors associated with cancer such as p53, CD40 ligand or feline IL-12 have been tested with success for treatment of different canine and feline tumors [26,27,28]. 

**Table 1:** Oncolytic viruses tested for canine and/or feline cancer therapy.

Virus strain	Virus family/Virus type	Study/Tumor type/Animal model	Ref.
Canine adenovirus type 2 (CAV2)	Adenoviridae (double stranded DNA viruses)	Infection of canine osteosarcoma cells and osteosarcoma xenografted mice	[29]
Human adenovirus type 5 (Ad5)	Adenoviridae	Infection of canine osteosarcoma, melanoma and mammary carcinoma cells	[24]
Ad5, CAV2	Adenoviridae	Infection of canine cells and osteosarcoma xenografted mice	[25]
CAV2	Adenoviridae	Infection of canine osteosarcoma cells and healthy dogs	[30]
CAV2	Adenoviridae	Treatment of canine osteosarcoma xenografts using tumor cells as a carrier for CAV2	[31]
Ad5-based vector with CD40 ligand (AdCD40L)	Adenoviridae	Treatment of canine malignant melanoma patients	[32,33]
Ad5-based vector encoding IL-12 (Ad hsp feline IL-12)	Adenoviridae	Treatment of cats with soft tissue sarcoma	[28]
Ad5-vector-mediated p53 gene transfer	Adenoviridae	Treatment of canine osteosarcoma xenografts	[26]
Canine Distemper Virus (CDV)	Paramyxoviridae (single stranded RNA viruses)	Infection of canine lymphoid, osteosarcoma and melanoma cells	[34]
Reovirus	Reoviridae (double stranded RNA viruses)	Infection of canine mast cell tumor cells (MCT) and treatment of MCT xenograft mice	[35]
Vaccinia virus (Lister) strain (GLV-1h68)	Poxviridae (double stranded DNA viruses)	Treatment of canine mammary adenoma and carcinoma and soft tissue sarcoma xenograft mice	[36,37,38]
Vaccinia virus (Lister) strain expressing anti-VEGF antibody (GLV-1h109)	Poxviridae	Treatment of canine soft tissue sarcoma and prostate xenograft mice	[39]
Vaccinia virus (Lister) strain (LIVP 6.1.1)	Poxviridae	Treatment of canine soft tissue sarcoma and prostate xenografted mice	[40]
Vaccinia virus (Lister) strain expressing anti-VEGF antibody (GLV-5b451)	Poxviridae	Treatment of feline mammary carcinoma xenograft mice	[41]
Myxoma virus (MYXV)	Poxviridae	Infection of different canine tumor cells	[42]
Myxoma virus (MYXV)	Poxviridae	Infection of feline carcinoma cells	[43]
Canary pox virus expressing IL2 (ALVAC-fIL2)	Poxviridae	Therapy of cats with feline fibrosarcomas	[14]
Vaccinia virus (Copenhagen) strain expressing IL2 (NYVAC-fIL2)	Poxviridae	Therapy of feline fibrosarcoma patients	[14]

Abbreviations: Ad5—Human adenovirus type 5; CAV2—Canine adenovirus type 2; IL2—interleukin 2; IL-12—interleukin 12; fIL2—feline interleukin 2; VEGF—Vascular endothelial growth factor.

However, the level of human adenovirus replication in canine cells is about 2 logs lower than in human cells [24]. On the other hand, canine adenovirus, while not as well studied, displays significantly improved viral replication in canine cancer cells. The conditionally replicating canine adenovirus 2 (CAV2) with the osteocalcin promoter showed replication in canine osteosarcoma cells only [30] and a significant therapeutic effect in canine osteosarcoma xenografts [29,31]. In addition, administration of this modified canine adenovirus to normal dogs showed only moderate virus-associated toxicity [30]. However, clinical use of CAV2 and its vectors can, under some conditions, be limited by pre-existing vector immunity [44]. Therefore, strategies to overcome the pre-existing humoral and cellular adenovirus specific immunity in dogs should be developed (see Section 3.2).

The introduction and expression of heterologous genes by adenoviruses can lead to enhanced therapeutic activity. For example, the adenoviral vector, AdCD40L, expressing a CD40 ligand exhibited complete tumor regression in 5 of 19 canine melanoma patients after intratumoral injection [32]. The results suggest that AdCD40L therapy is safe as administered and could have beneficial effects in treatment of canine melanoma.

### 2.2. Morbilliviruses

Canine distemper virus (CDV) is an enveloped virus with a single stranded RNA genome belonging to the genus *morbillivirus* of the *paramyxoviridae* family. It is a close relative of measles virus (MV). In fact, both MV and CDV use a similar cellular receptor for entry into cells [45]. Historically, children with Hodgkin’s disease were observed to experience regression after concurrent MV infection [46]. These observations prompted the consideration of attenuated MV for the treatment of human lymphoma and, consequently, measles virus has shown promising anti-tumor activity against a variety of malignant neoplasms in both preclinical and clinical studies [47]. Because of its similarity to MV, CDV was considered for treatment of canine lymphoma. CDV binds to the cellular receptor, the signaling lymphocyte activation molecule (SLAM or CD150) [48], which is over-expressed on malignant canine B and T lymphocytes [34]. Attenuated CDV was able to infect canine lymphoma cells in cell culture via binding to CD150 and to induce apoptosis in these cells [34]. While preliminary, these results support the continued evaluation of CDV for the treatment of canine lymphoma.

Nevertheless, the most dogs are vaccinated against canine distemper virus and the high prevalence of virus-neutralizing antibodies is one major obstacle to the use of CDV in canine clinical trials. The use of vectors of non-canine origin like e.g., MV or removing key neutralizing epitopes on the surface of viral capsid proteins might help to avoid pre-existing immunity. In addition, intratumoral or mucosal virus application or administration of higher virus doses could also be a solution for pre-existing immunity problems (for general review see [49]).

### 2.3. Reovirus

Reovirus, a non-enveloped icosahedral virus with segmented double stranded RNA, has been extensively tested in oncolytic virotherapy of human cancers over the past decade. While its clinical development has advanced into phase II and III clinical trials with human cancer patients, it has been hardly studied as a canine or feline oncolytic agent. A very recent study demonstrated for the first time that canine mast cell tumors (MCT) were highly susceptible to reovirus infection *in vitro* [35]. In addition, a single intratumoral reovirus injection significantly regressed canine mast cell tumor xenografts [35]. However, reovirus also infected normal canine mast cells raising safety concerns. Subsequent studies should address these concerns before using reovirus as an oncolytic agent for canine cancer therapy.

### 2.4. Poxviruses

Several preclinical and clinical studies with vaccinia virus (VACV) strains have demonstrated promising oncolytic properties [50]. VACV is a double stranded DNA virus of the poxvirus family [51,52]. Since it was the first widely used vaccine (in over 200 million people), resulting in worldwide eradication of smallpox, much is known about its safety profile [53]. Three different oncolytic vaccinia virus strains, GL-ONC1/GLV-1h68, V-VET-1/LIVP 6.1.1 (Genelux Corporation, San Diego, CA, USA) and JX-594 (Jennerex Biotherapeutics, Inc., San Francisco, CA, USA) are now undergoing clinical trials with either human or canine cancer patients [40,54,55]. The JX-594 construct was derived from the Wyeth strain by deletion of the thymidine kinase (*tk*) gene via insertion of GM-CSF and *lacZ* genes [56]. LIVP 6.1.1 was isolated from a wild type stock of Lister strain of vaccinia virus (Lister strain, Institute of Viral Preparations (LIVP), Moscow, Russia) and represents a “native” virus (no genetic manipulations were conducted). Interestingly, the thymidine kinase (*tk*) gene of LIVP 6.1.1 virus is inactive [57]. Several groups have reported that TK-mutant vaccinia viruses are significantly attenuated and demonstrate enhanced tumor-specific replication *in vivo* [54,58]. GLV-1h68 (named GL-ONC1 as produced for clinical investigation) was developed from the Lister strain by inserting three expression cassettes encoding *Renilla* luciferase–*Aequorea* green fluorescent protein fusion (Ruc-GFP), LacZ, and β-glucuronidase into the *F14.5L*, *J2R* (thymidine kinase) and *A56R* (hemagglutinin) loci of the viral genome, respectively [54]. 

GLV-1h68 has been tested with success in the treatment of canine mammary adenoma, mammary carcinoma and soft tissue sarcoma in xenograft tumor models [36,37,38]. In all three models, significant inhibition of tumor growth was observed after a single systemic administration of GLV-1h68 [36,37,38]. Additionally, GLV-1h68 enabled the detection of metastases via optical imaging [59,60]. GLV-1h109, a variant of GLV-1h68, expressing the anti-VEGF (Vascular Endothelial Growth Factor) single chain antibody (scAb) GLAF-1, demonstrated strong antitumor effects in canine soft tissue sarcoma and prostate carcinoma xenograft models [39]. Localization of GLV-1h109 and expression of GLAF-1 in the tumor tissue inhibited tumor angiogenesis. Similar data was obtained by the use of the new recombinant oncolytic vaccinia virus strain GLV-5b451, a variant of LIVP 6.1.1, expressing GLAF-2, in a feline mammary carcinoma xenograft model [41]. GLAF-1 and GLAF-2 single chain antibodies are identical with exception that GLAF-1 contains a FLAG-tag for purification purposes [61]. 

The oncolytic effect of LIVP 6.1.1 was studied in a panel of four different canine cancer cell lines including soft tissue sarcoma STSA-1, melanoma CHAS, osteosarcoma D17 and prostate carcinoma DT08/40 cells [40]. LIVP 6.1.1 was shown to efficiently infect, replicate in and lyse all tested canine cancer cells in culture [40]. In addition, two subclones of LIVP, LIVP 6.1.1 and LIVP 1.1.1, have shown great promise in the treatment of STSA-1 and/or DT08/40 tumor xenografts [38,40]. Interestingly, DNA sequence analysis of LIVP 1.1.1 revealed that the thymidine kinase (tk) gene of this strain is also inactive [57].

A phase I clinical study is underway to evaluate the safety of intravenous administration of LIVP 6.1.1 for treatment of canine cancer patients at Angel Care Cancer Center, Carlsbad, CA, USA. 

Oncolytic activity of several other poxviruses, such as ALVAC, NYVAC and myxoma virus, has been evaluated in canine and feline tumors. Despite the fact that myxoma virus (MYXV) replicates only in lagomorphs and is only pathogenic in the European rabbit, it has shown efficient infection in human cancer cell types. Constant activation of cellular transformation pathways and the inability of tumor cells to elicit antiviral immune response helps MYXV to replicate in human cancer cells [62,63]. Moreover, MYXV can infect and lyse various canine and feline tumor cells *in vitro* [42,43]. Further studies of myxoma virus as an oncolytic agent *in vivo* are needed to determine its potential in treating dog and cat cancers.

ALVAC-fIL2 and NYVAC-fIL2 are recombinant, non-replicating canary poxvirus and highly attenuated vaccinia virus, respectively, encoding the feline IL2 gene [14]. When combined with surgery and radiotherapy, treatment with either of these viruses prevented feline fibrosarcoma recurrence in cats [14]. 

### 2.5. What Could Be the Possible Mechanisms of Oncolytic Virus Mediated Tumor Ablation?

**Figure 1:**
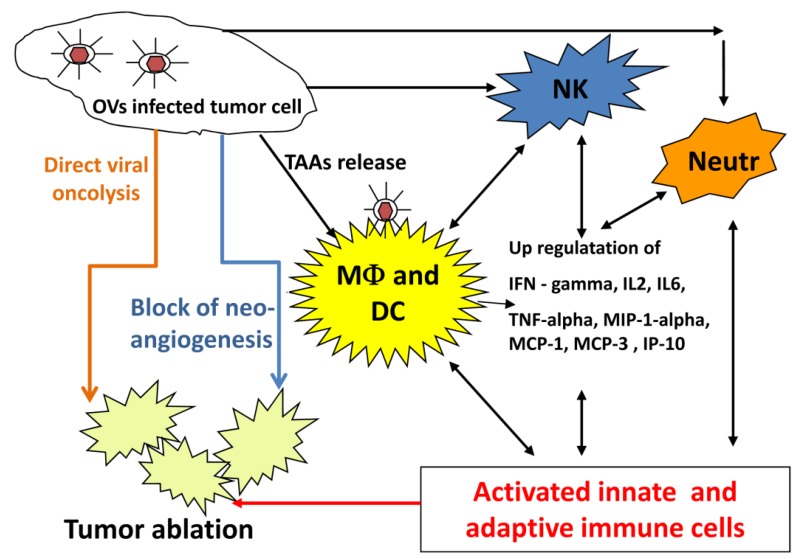
Possible mechanisms of oncolytic virus mediated tumor ablation.

Abbreviations: MФ: macrophages; NK: natural killer cells; DC: dendritic cells; Neutr.: neutrophils; IFN-gamma: interferon-gamma; IL-2: interleukin-2: IL-6: interleukin-6; TNF-alpha: tumor necrosis factor alpha: IP-10: interferon gamma-induced protein 10; MIP-1 alpha: macrophage inflammatory protein-1; MCP-1: monocyte chemotactic protein-1; MCP-3: monocyte chemotactic protein-3; TAA: tumor-associated antigens.

An oncolytic virus destroys tumors either by direct viral lysis of tumor cells [64,65], by destruction of the tumor vasculature [66], by induction of host antitumoral immune responses [67,68,69], or most likely, a combination of these mechanisms [11,70] (Figure 1). An increased infiltration of neutrophils, macrophages and natural killer (NK) cells to the tumor site might be involved in the VACV-mediated immune response in different canine cancer xenograft models [37,38,40]. The presence of such activated inflammatory cells in the tumor tissue may enhance the antitumoral effect by increasing the phagocytic or cytotoxic activities of these cells [71,72]. In addition, an increase in proinflammatory interferon-gamma (IFN-gamma), interleukin-2 (IL-2), interleukin-6 (IL-6), tumor necrosis factor alpha (TNF-alpha), interferon gamma-induced protein 10 (IP-10), macrophage inflammatory protein-1 alpha (MIP-1 alpha), macrophage inflammatory protein-1 beta (MIP-1 beta), monocyte chemotactic protein-1 (MCP-1), and monocyte chemotactic protein-3 (MCP-3) was observed in vaccinia virus infected canine xenografted mice [37,38]. Many of these proteins stimulate innate immunity mediated by dendritic cells, neutrophils, macrophages and NK cells. 

OVs naturally prevent neoangiogenesis either by direct infection and destruction of tumor vasculature [66] or “vascular normalization” in tumor tissue, as described by Winker and colleagues [73]. Moreover, oncolytic viruses can be additionally armed to enhance their natural antiangiogenic ability. VEGF is a key regulator of tumor angiogenesis and several anti-VEGF strategies have been developed for the treatment of different cancers [74,75,76]. Vaccinia virus expressing anti-VEGF antibodies significantly decreased neoangiogenesis at the tumor site and inhibited tumor growth in canine and feline xenografts [39,41]. Thus, strategies employing virus-encoded and delivered anti-VEGF antibodies in combination with OV may be effective therapeutic approaches for pet cancer patients.

At the present there are not enough data from pet cancer patients treated with oncolytic viruses to allow an understanding of OV-mediated tumor ablation. However, it is expected that factors such as origin of tumor, stage of tumor development and combination therapy, and, importantly, the balance between antiviral and antitumoral immune responses will play a role.

## 3. Open Questions Regarding the Use of Oncolytic Virotherapy in Veterinary Medicine

The major obstacles that restrict the optimal use of oncolytic viruses as therapeutics for canine or feline cancer patients are viral toxicity, ineffective delivery of virus to tumor and inefficient spread of OVs throughout the tumor mass. 

### 3.1. Toxicity of Oncolytic Viruses to Pet Cancer Patients

As for all experimental therapeutic agents and treatments, a primary concern of virotherapy is toxicity. Treatment-related toxicity is common for chemotherapy and radiation therapy in canine or feline cancers [77,78,79]. While only a few clinical trials of oncolytic virotherapy in canine or feline cancer patients have been carried out, evidence for the lack of toxicity of OVs in pets is emerging. Intravenous administration of canine adenovirus-2 in normal dogs and AdCD40L (adenovector expressing the CD40 ligand) in canine malignant melanoma cases did not show any virus associated toxicity [30,80]. In a very recently completed phase I study, Westberg and colleagues reported that AdCD40L therapy in canine melanoma patients was safe and only mild adverse effects were associated with treatment [32]. Sufficient evidence of oncolytic viruses as a safe therapeutic modality is displayed by several studies [33,81,82]. Furthermore, genetic engineering of oncolytic viruses is supported by the safety data from genetically engineered products like DNA vaccine in dogs and non-human primates [83,84,85]. Overall both wild type and genetically engineered oncolytic virus therapy appears to be as safe as standard anti cancer therapies. 

### 3.2. Optimization of OV Delivery to the Tumor Tissue and Metastases

Immune responses against therapeutic viruses may presumably hamper ongoing viral replication in immunocompetent dogs and cats. Several factors including neutralizing antibodies, inactivation by complement and sequestration in the liver and spleen could impair the effectiveness of the virus especially after intravenous virus application, the preferred route of administration for treatment of metastatic cancer. One of the most promising strategies to evade virus inactivation by these factors is carrier-based delivery. Several types of cells, such as immune cells [86], stem cells [87] and tumor cells [88] have been successfully utilized as carriers of OVs to the tumor. 

Canine osteosarcoma cells treated with replication selective canine adenovirus (OCCAV) were used as virus carriers for evading pre-existing neutralizing antibodies against adenovirus. When administered systemically, even in the presence of adenovirus neutralizing antibodies, OCCAV carrier cells showed superior infection of tumors and tumor regression in a xenograft mouse model compared to OCCAV alone [31]. Moreover, the enhanced oncolytic effects were attributed to an increase in the effective local viral dose in the tumor as a consequence of the tumor-specific delivery of the virus by the cells and the escape of the pre-existing antiviral immunity [88,89]. 

In addition, virus coatings with biocompatible polymers such as polyethylene glycol (PEG) [90], silk-elastinlike protein [91] or *N*-[2-hydroxypropyl]meth-acrylamide (HPMA) [92] or with serum proteins [93,94] showed a minimized sequestration by the mononuclear phagocytic system in the liver and spleen. Furthermore, delivery of viruses within injectable polymer matrices has been shown to enhance antitumor effects compared to virus alone. Intratumoral injection of an adenovirus vector into human head and neck cancer xenograft tumors in a silk-elastinlike protein hydrogel increased the stability of the virus and the duration of its release into the tumor microenvironment [67].

In general, carrier-mediated deliveries of OVs may increase the efficiency of viral tumor colonization and protect the virus against the components of the host innate or acquired immune system. 

### 3.3. Enhancing Viral Spread throughout the Tumor

The improvement of virus spread in tumor tissue is an important challenge for effective oncolytic virotherapy [95]. The tumor extracellular matrix (ECM) or stroma presents a substantial physical barrier to virus spread. Structural components of tumor ECM, such as collagens and proteoglycans, have been shown to hinder distribution of large therapeutic molecules and viruses [96]. Several groups have reported that the intratumoral spread and efficacy of OVs was improved by protease or hyaluronidase-mediated digestion of tumor ECM [97,98,99]. While preliminary, these results suggest that such strategies may be clinically useful. 

### 3.4. Biosafety of Treatment

Oncolytic viruses also raise new biosafety and risk management issues [100,101]. The risk assessment for trials with these agents must take into account and mitigate the potential risk of transmission of the infectious agent to other pets and persons in contact with the treated patient. The zoonotic aspects or risk to pet owners and general public has to be monitored. The spectrum of diseases caused by parental viral strains in dogs or cats is an important safety factor for consideration. If necessary, the risk of disease or adverse effects from a viral therapeutic could be countered with antiviral agents effective against the viral strains considered for cancer treatment. 

## 4. Conclusions

The significant incidence and mortality associated with canine and feline cancers continues to challenge modern veterinary medicine to develop more reliable therapies. One of the most promising novel cancer therapies is oncolytic virotherapy. This method is based on the capability of OVs to preferentially infect and lyse cancer cells and to initiate tumor-specific immunity. Several oncolytic viruses including human and canine adenoviruses, canine distemper virus (CDV), reovirus and vaccinia virus strains have been tested with convincing results in preclinical studies. 

As for oncolytic virotherapy of human cancers, the most important challenges for the successful clinical use of OVs in veterinary practice are reduction of viral toxicity, optimization of virus delivery to tumor, and enhancement of viral spread throughout the tumor mass. 

Recently, the first clinical studies with vaccinia and adenovirus for canine cancer therapy are underway and we look forward to the forthcoming demonstrations of clinical utility.
